# Evaluation of the Membrane Damage Mechanism of Chlorogenic Acid against *Yersinia enterocolitica* and *Enterobacter sakazakii* and Its Application in the Preservation of Raw Pork and Skim Milk

**DOI:** 10.3390/molecules26216748

**Published:** 2021-11-08

**Authors:** Lu Tian, Mi Wu, Wenyao Guo, Hui Li, Zhongchao Gai, Guoli Gong

**Affiliations:** School of Food and Biological Engineering, Shaanxi University of Science and Technology, Xi’an 710021, China; tianlu@sust.edu.cn (L.T.); 1904091@sust.edu.cn (M.W.); 1804063@sust.edu.cn (W.G.); lihui@sust.edu.cn (H.L.); zhongchaogai@sust.edu.cn (Z.G.)

**Keywords:** chlorogenic acid, *Y. enterocolitica*, *E. sakazakii*, antibacterial mechanism, raw pork, skim milk, response surface methodology

## Abstract

Plant-derived antimicrobial agents have adequate antimicrobial effects on food-borne pathogens, which can be used as food preservatives. The purpose of this study was to evaluate the antibacterial mechanism of chlorogenic acid (CA) against *Yersinia enterocolitica* and *Enterobacter sakazakii*. The minimum inhibitory concentration (MIC) of CA was determined by employing the broth microdilution method. Then, the cell function and morphological changes of *Y. enterocolitica* and *E. sakazakii* treated with CA were characterized. Finally, the growth inhibition models of *Y*. *enterocolitica* in raw pork and *E. sakazakii* in skim milk were constructed through the response surface methodology. The results demonstrated that CA has a satisfactory inhibitory effect against *Y. enterocolitica* and *E. sakazakii* with a MIC of 2.5 mg/mL. In addition, CA inhibited the growth of *Y. enterocolitica* and *E. sakazakii* via cell membrane damage, such as depolarization of the cell membrane, reduction in intracellular adenosine triphosphate (ATP) and pH levels, and destruction of cell morphology. Moreover, CA reduced two log cycles of *Y. enterocolitica* in raw pork and *E. sakazakii* in skim milk at a certain temperature. According to the corresponding findings, CA has the potential to be developed as an effective preservative to control *Y. enterocolitica* and *E. sakazakii*-associated foodborne diseases.

## 1. Introduction

Due to the development of society, as well as the continuous improvement of living standards, food safety has garnered increased attention; meanwhile, foodborne diseases serve as one of the main factors affecting food safety [1]. Foodborne diseases are mainly caused by foodborne pathogens, which seriously threaten human health and affect the development of the social economy. The Centers for Disease Control and Prevention estimates that 48 million illnesses have been caused by foodborne diseases, incurring annual economic costs to the U.S. estimated at USD 152 billion to USD 1.4 trillion [2]. *Yersinia enterocolitica*, a kind of gram-negative bacterium, is widely distributed in foods such as vegetables, meat, dairy, and aquatic products [3]. *Y. enterocolitica* can proliferate at 4 °C, making it dangerous if contaminated food is stored under refrigerated conditions. Pork is a common host of *Y. enterocolitica*, and most human infections with *Y. enterocolitica* are caused by eating raw or undercooked contaminated pork [4]. *Y. enterocolitica* is mainly transmitted through food or water sources, causing fever, enterocolitis, and sepsis [5]. Therefore, it is of great significance to prevent the contamination of foodborne pathogens during the process of food processing, packaging, storage, and transportation.

*Enterobacter sakazakii*, a type of food-borne pathogenic bacteria, is widely presented in soil, water, milk products, and vegetables, and its pollution pathways are very extensive [6]. *E. sakazakii* can cause bacteremia, necrotizing enterocolitis, and neonatal meningitis, which has a case fatality rate of 50–80%. *E. sakazakii* possesses a certain level of heat and drying resistance; hence, it can survive in environments with low water activity, as well as in milk powder for two or more years [7]. In order to meet the needs of consumers in regard to food safety, nutritional, value, and sensory characteristics, a variety of food preservation technologies have been developed. The heat treatment technology of food involves sterilizing the food at a certain temperature to extend the shelf life of the food. However, overheating will contribute to the sensory and nutrition properties of the food [8]. In addition, chemical preservatives are widely used to extend the shelf life of food. However, chemical preservatives can easily lead to pathogen resistance, environmental pollution, and bring about toxic side-effects, which directly threaten the health of consumers [9]. Today, consumers tend to pursue natural and nontoxic foods. As a result, plant-derived preservatives are more popular due to their good antibacterial effects and low toxicity characteristics [10].

Plant-derived preservatives refer to substances extracted from plants that possess satisfactory antibacterial properties [11]. Plant-derived preservatives have antibacterial and food preservation effects, as well as anti-oxidation, anti-aging, and other pharmacological properties [12], which agree with the concepts of naturality, greenness, safety, and health in terms of consumption. Chlorogenic acid (CA) is a phenolic acid formed by caffeic acid and quinic acid [13]. It widely exists in natural plants such as honeysuckle and eucommia ulmoides. Studies have reported that CA has a wide range of pharmacological effects, which is internationally known as plant gold [14]. CA has been found to prevent lipid oxidation in food; the oxidation of lipids can lead to undesirable off-flavors and may also influence food quality parameters such as texture, taste, and nutritional profile [15]. Previous studies have demonstrated that CA has adequate inhibitory effects on *P. aeruginosa*, *E. coli*, and *S. aureus* [16]. Li et al. reported that CA effectively inhibits the growth of *S. aureus* by destroying the cell membrane [17]. However, the antibacterial mechanism of CA against *Y. enterocolitica* and *E. sakazakii* has yet to be described.

Therefore, in this study, the antibacterial activity of CA against *Y. enterocolitica* and *E. sakazakii* was evaluated by measuring the MIC and growth curve. The effects of CA on the cell membrane permeability of *Y. enterocolitica* and *E. sakazakii* were analyzed by measuring the membrane potential level, intracellular ATP, and intracellular pH (pHin) level. The effects of CA on the cell membrane integrity and cell morphology of *Y. enterocolitica* and *E. sakazakii* were then observed using a confocal laser scanning microscope (CLSM) and field emission gun scanning electron microscope (FEGSEM). Finally, the growth inhibition models of *Y. enterocolitica* in raw pork and *E. sakazakii* in skim milk were constructed using the response surface methodology.

## 2. Results and Discussion

### 2.1. MIC of CA on Y. enterocolitica and E. sakazakii

**Figure 1:**
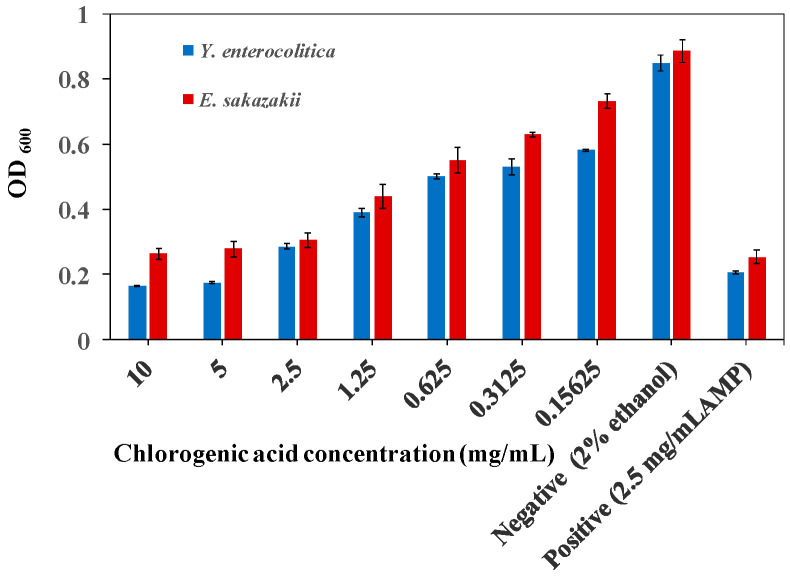
The OD_600_ of *Y. enterocolitica* and *E. sakazakii* cells treated with different concentrations of CA at 8 h.

CA was found to have good antibacterial effects against *Y. enterocolitica* and *E. sakazakii*, which increased with CA concentration (Figure 1). When the concentration of CA was 2.5 mg/mL, the growth of *Y. enterocolitica* and *E. sakazakii* was inhibited, and the cell density was 0.28 and 0.30, respectively, which was almost the same as the positive control group. These findings indicated that the MIC of CA on *Y. enterocolitica* and *E. sakazakii* was 2.5 mg/mL.

### 2.2. Effect of CA on Growth Curve of Y. enterocolitica and E. sakazakii

**Figure 2:**
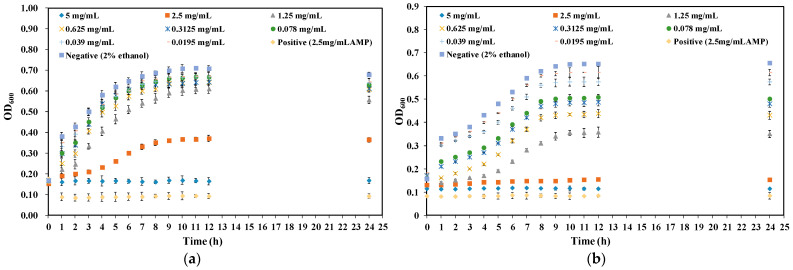
Effect of CA on the growth curve of (**a**) *Y. enterocolitica* and (**b**) *E. sakazakii*.

In order to further analyze the antibacterial activity of CA against *Y. enterocolitica* and *E. sakazakii*, growth curves of *Y. enterocolitica* and *E. sakazakii* treated with different concentrations of CA were then established. The growth trend of *Y. enterocolitica* treated with CA lower than 1/8 MIC was basically the same as that of the control group, indicating that CA had no obvious influences on the growth of *Y. enterocolitica* (Figure 2a). However, when the concentration of CA reached MIC and 2 MIC, the cell density of *Y. enterocolitica* did not change within 24 h. The corresponding results confirmed that higher concentrations of CA led to a longer lag phase and lower growth rate of *Y. enterocolitica*.

*E. sakazakii* in the negative control group began to enter the log phase at 3 h and reached the stable phase at 10 h (Figure 2b). The growth trend of *E. sakazakii* treated with CA at a lower MIC was basically found to be the same as that of the control group; however, when *E. sakazakii* treated with CA at MIC and 2 MIC, bacterial growth was slow, and the lag phase was prolonged. The results demonstrated that CA had a good inhibitory effect on the growth of *Y. enterocolitica* and *E. sakazakii*; specifically, it performed better on *Y. enterocolitica*.

### 2.3. Effect of CA on Membrane Potential of Y. enterocolitica and E. sakazakii

The potential difference between two sides of the cell membrane is referred to as the membrane potential, which is an electrical phenomenon accompanied by cell life activities [18]. Maintaining normal membrane potential is vital for ATP synthesis, as well as the normal physiological function of cells. DiBAC4(3) is a lipophilic anionic fluorescent dye, and it is sensitive to cell membrane potential [19], which is commonly used as an indicator dye for cell membrane potential. The cell is stimulated by the external environment, and the cell membrane is in different potential states. When fluorescence intensity decreases, it indicates that the cell is hyperpolarized; on the contrary, increased fluorescence intensity points to cell depolarization [20].

**Figure 3:**
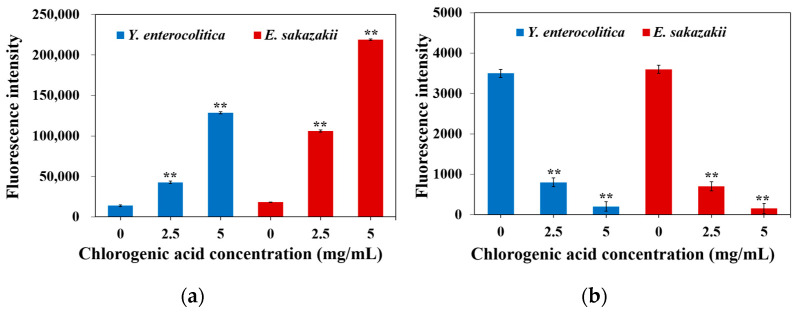
Effect of CA on the (**a**) membrane potential level and (**b**) intracellular ATP level of *Y. enterocolitica* and *E. sakazakii*. Error bars represent the standard deviation (*n* = 3). ** *p* ≤ 0.01 represent significant differences between the treatment group and control group.

The results showed that CA significantly increased the membrane potential of *Y. enterocolitica* and *E. sakazakii* (Figure 3a), signifying that the cell membranes of *Y. enterocolitic*a and *E. sakazakii* were depolarized. The change in cell membrane potential affects the power of protons in and out of the cell and inhibits the synthesis of ATP, eventually leading to bacterial death. Similar studies have shown that ginger essential oil depolarizes the cell membranes of *S. aureus* and *E. coli* [21]. Depolarization and hyperpolarization occur mainly due to pH changes or increased ion movement, especially the diffusion of K^+^ or K^+^ with several other ions. They diffuse inward and outward to balance the membrane potential. In addition, maintaining ion homeostasis is an integral part of cell growth, which is essential for many energy-related metabolism mechanisms [22]. Therefore, the perturbation of membrane potential caused by CA may affect cell metabolism and eventually lead to bacterial death.

### 2.4. Effect of CA on Intracellular ATP of Y. enterocolitica and E. sakazakii

During energy conversion and transmission, ATP serves a key substance and directly provides energy for cell life activities (respiration, proliferation, differentiation, and apoptosis) [23]. The ATP levels in intact cells are in a stable state. However, the destruction of cell homeostasis and integrity may cause changes in intracellular ATP concentrations under stress [24]. The change in ATP content is related to the energy metabolism of various organs. Therefore, influencing the synthesis of ATP is a way for natural preservatives to exert their antibacterial effect.

The effect of CA on the intracellular ATP content of *Y. enterocolitica* and *E. sakazakii* is shown in Figure 3b. In the control group, the intracellular ATP contents of *Y. enterocolitica* and *E. sakazakii* were found to be 3500 and 3600, respectively, while the intracellular ATP contents of *Y. enterocolitica* and *E. sakazakii* treated with CA were noted to decrease significantly. ATP is mainly produced in the mitochondria, which indicates that CA may promote the accumulation of reactive oxygen species (ROS) and lead to the damage of pathogenic mitochondria, resulting in the decline in pathogenic activity. Guo et al. found that luteolin sharply decreased the intracellular ATP concentration of *Trueperella pyogenes* [25]. The decrease in intracellular ATP concentration may be caused by the rise in the ATP hydrolysis rate by the proton pump, resulting in the rapid consumption of ATP; alternatively, the change in cell membrane permeability may result in ATP leakage.

### 2.5. Effect of CA on Intracellular pH of Y. enterocolitica and E. sakazakii

As intracellular ATP depletion and decreased membrane potentials after CA treatment suggest the effects of CA on the cellular membrane, pHin was comprehensively investigated in this study. Once the CFDA-SE fluorescent probe enters the cell, it is hydrolyzed by esterase into carboxyfluorescein succinimidyl ester (CFSE), which emits fluorescence and stays there [26]. Therefore, based on the fluorescence response to pH, the pHin changes were monitored in living cells.

**Figure 4:**
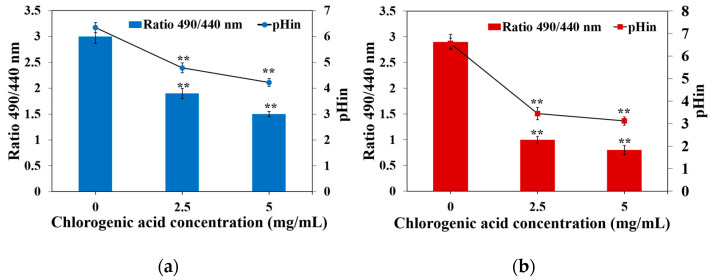
Effect of CA on the intracellular pH level of (**a**) *Y. enterocolitica* and (**b**) *E. sakazakii*. Error bars represent the standard deviation (*n* = 3). ** *p* ≤ 0.01 represent significant differences between the treatment group and control group.

The pHin of *Y. enterocolitica* and *E. sakazakii* treated with CA was observed to decrease significantly (*p* < 0.01). Specifically, the pHin of *Y. enterocolitica* and *E. sakazakii* without CA treatment was found to be 6.33 ± 0.13 and 6.57 ± 0.15, respectively. After being treated with CA at MIC, the pHin of *Y. enterocolitica* and *E. sakazakii* decreased to 4.70 ± 0.10 and 3.44 ± 0.06, respectively. After treatment with CA at 2 MIC, the pHin of *Y. enterocolitica* and *E. sakazakii* decreased to 4.22 ± 0.05 and 3.12 ± 0.09 (Figure 4a,b). pHin has been reported to play an essential role in the transcription and synthesis of bacterial DNA, enzyme activity, and protein synthesis [27]. In addition, pHin also controls the cell membrane. The change in pHin means that the permeability of the cell membrane has changed. CA can reduce the level of pHin of *Y. enterocolitis* and *E. sakazakii*, demonstrating that CA can change the internal environment of the normal growth of *Y. enterocolitis* and *E. sakazakii*, leading to bacterial death. Consistently, a study by Li et al. reported that a tannin-rich fraction from pomegranate rind significantly decreased the pHin of *L. monocytogenes* [28].

### 2.6. Effect of CA on Cell Membrane Damage of Y. enterocolitica and E. sakazakii

The cell membrane is one of the main components of the cellular structure, which is involved in maintaining cell integrity, material transportation, receptor function, and information transmission [29]. The LIVE/DEAD BacLight™ Bacterial Viability Kit was used to evaluate the effect of CA on the cell membrane integrity of *Y. enterocolitica* and *E. sakazakii*. SYTO 9 can enter all cells, which binds to DNA and RNA, emitting green fluorescence. PI can only enter cells with damaged cell membranes, emitting red fluorescence. Moreover, sublethal injured cells with damage and esterase activity emit yellow fluorescence [30]. In this experiment, the fluorescence color of *Y. enterocolitica* and *E. sakazakii* after double staining with SYTO 9/PI was observed by the merged images of CLSM, to show the effect of CA on the cell membrane integrity of *Y. enterocolitica* and *E. sakazakii*.

**Figure 5:**
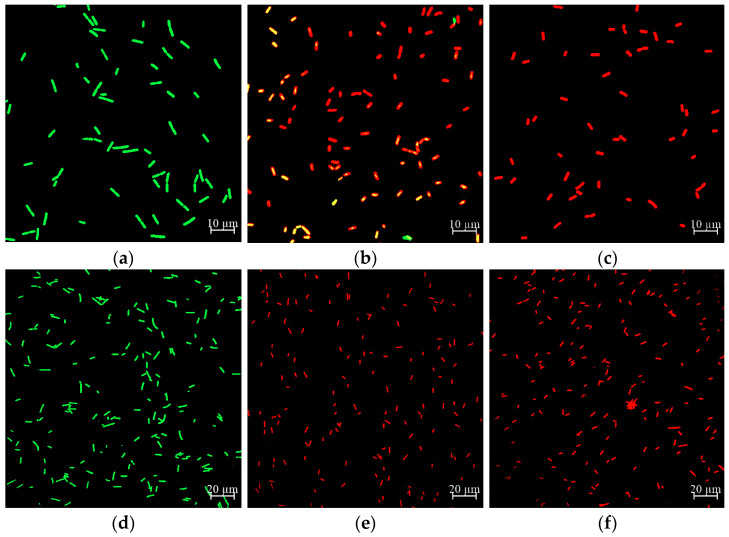
Effect of CA on the cell membrane integrity of *Y. enterocolitica* and *E. sakazakii*. (**a**) Untreated *Y. enterocolitica*, (**b**) *Y. enterocolitica* treated with MIC CA, (**c**) *Y. enterocolitica* treated with 2 MIC CA, (**d**) untreated *E. sakazakii*, (**e**) *E. sakazakii* treated with MIC CA, and (**f**) *E. sakazakii* treated with 2 MIC CA.

The results showed that the cells of *Y. enterocolitica* and *E. sakazakii* without CA treatment emitted green fluorescence (Figure 5a,d), indicating that the cell membrane was intact and undamaged. After treatment with CA at MIC, most *Y. enterocolitica* cells exhibited red fluorescence; however, some cells gave off yellow fluorescence (Figure 5b), indicating that part of the cell membrane was damaged. However, all *E. sakazakii* cells displayed red fluorescence (Figure 5e), indicating that all cell membranes were damaged. After being treated with CA at 2 MIC, all cells of *Y. enterocolitica* and *E. sakazakii* showed red fluorescence (Figure 5c,f), indicating that all cell membranes were destroyed. Similarly, Su et al. put forward that the cell membrane of *P. aeruginosa* became damaged following CA treatment, and the number of membrane damaged cells increased with treatment time and CA concentration [31].

### 2.7. Effect of CA on Cell Morphology of Y. enterocolitica and E. sakazakii

**Figure 6:**
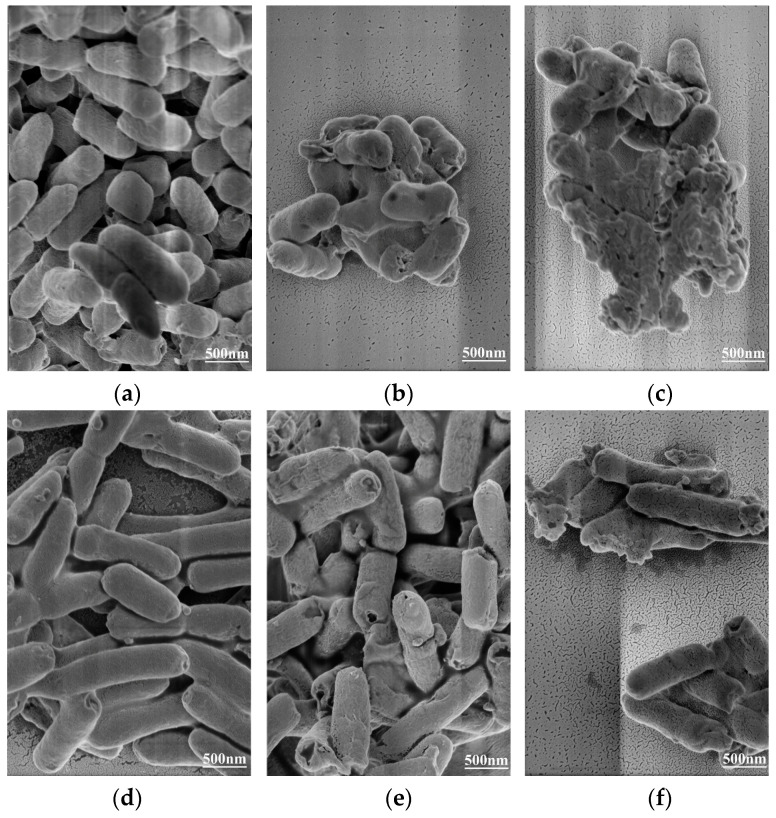
Effect of CA on cell morphology of *Y. enterocolitica* and *E. sakazakii*. (**a**) Untreated Y. enterocolitica, (**b**) *Y. enterocolitica* treated with MIC CA, (**c**) *Y. enterocolitica* treated with 2 MIC CA, (**d**) untreated *E. sakazakii*, (**e**) *E. sakazakii* treated with MIC CA, and (**f**) *E. sakazakii* treated with 2 MIC CA.

Electron microscopy is a powerful tool, which is used to better understand the influence of external factors on cell morphology. In this study, FEGSEM was used to observe the changes in cell morphology of *Y. enterocolitica* and *E. sakazakii* treated with CA. The degree of damage of *Y. enterocolitica* and *E. sakazakii* cells was shown to increase with the rise in CA concentration. The cells of *Y. enterocolitica* and *E. sakazakii* without CA treatment were complete, and the cells were rod-shaped, full, and smooth with obvious edges (Figure 6a,d), indicating that 2% ethanol had no effect on their cell morphology. When *Y. enterocolitica* and *E. sakazakii* were treated with CA at MIC, the bacterial surface was uneven and shrunk, and some cells appeared to adhere to each other (Figure 6b,e). After *Y. enterocolitica* and *E. sakazakii* were treated with CA at 2 MIC, the bacteria were seriously damaged, and the inherent corynebacterium morphology of *Y. enterocolitica* and *E. sakazakii* was lost, with a large number of contents being leaked (Figure 6c,f). These observations showed that CA induced morphological changes in *Y. enterocolitica* and *E. sakazakii* in a concentration-dependent manner.

In this study, the morphological changes of the two bacteria may be caused by the effect of CA on the integrity and permeability of the cell membrane, which led to the separation of the cell membrane from the cell wall, as well as the leakage of intracellular material. These findings were consistent with the results of the membrane potential measurement, indicating that the cell membrane may serve as the main target for the antibacterial effects of CA. Severe membrane damages due to cell permeability result in a bacterial morphological transition from rod-shaped cells to amorphous cells, which is consistent with FEGSEM observations of *Shigella dysentery* and *Streptococcus pneumoniae* treated with CA. Lou et al. demonstrated that CA significantly increased the permeability of the outer membrane and plasma membrane of *Shigella dysentery* and *Streptococcus pneumoniae*, resulting in the loss of barrier function, as well as the slight leakage of nucleotides [32]. Citrus essential oil can also change the permeability and integrity of the plasma membrane of *E. coli* and *L. rhamnosus*, thus achieving antibacterial effects [33].

### 2.8. Inhibitory Effect of CA on Y. enterocolitica in Raw Pork and E. sakazakii in Skim Milk

Gram-negative bacteria *Y. enterocolitica* and *E. sakazakii* are common pathogens, which are found in raw pork and skim milk, respectively. The effect of CA on the growth of *Y. enterocolitica* in raw pork and *E. sakazakii* in skim milk was evaluated according to the count of viable bacteria. Positive values represented reduction while negative values represented promotion (Table 1 and Table 2). According to the standard deviation in parentheses next to the *Y* value, the difference between the parallel samples was found to be very small. The number of bacteria of *Y. enterocolitica* and *E. sakazakii* in the untreated group increased by 2–3 log cycles, which was positively correlated with CA concentration and temperature. In addition, when *Y. enterocolitica* and *E. sakazakii* were treated with CA at MIC, the growth of *Y. enterocolitica* in raw pork and *E. sakazakii* in skim milk was almost completely inhibited. Moreover, when *Y. enterocolitica* and *E. sakazakii* were treated with CA at 2 MIC, the growth of *Y. enterocolitica* in raw pork and *E. sakazakii* in skim milk was inhibited by 1.7–2 log cycles. Therefore, CA was shown to effectively inhibit the growth of *Y. enterocolitica* in raw pork and *E. sakazakii* in skim milk. Using Design Expert 8.06 software to build the following quadratic model, the regression equations of *Y. enterocolitica* and *E. sakazakii* were: Y = −1.6771 − 0.0701 *X*_1_ − 0.0210 *X*_2_ + 1.2490 *X*_3_ + 0.0001 *X*_1_*X*_2_ + 0.0043 *X*_1_*X*_3_ − 0.0012 *X*_2_*X*_3_ + 0.0014 *X*_12_ + 0.0001 *X*_22_ − 0.0790 *X*_32_ *and Y =* −1.4329 − 0.0809 *X*_1_ − 0.0329 *X*_2_ + 1.2847 *X*_3_ + 0.0001 *X*_1_*X*_2_ + 0.0035 *X*_1_*X*_3_ − 0.0012 *X*_2_*X*_3_ + 0.0017 *X*_12_ + 0.0002 *X*_22_ − 0.0766 *X*_32_, where *Y* represents the logarithm of *Y. enterocolitica* and *E. sakazakii* cell inhibition; and *X*_1_, *X*_2_, and *X*_3_ refer to time, temperature, and CA concentration, respectively.

In order to test the validity of the regression equation and evaluate the associations of *Y. enterocolitica* and *E. sakazakii* inhibition with the various factors, the regression equation was analyzed by variance analysis (Table 3 and Table 4). Accordingly, an overall association was presented between them, with the correlation coefficient R-Squared being 0.9986 and 0.9981, while the correction coefficient Adj R-Squared was 0.9925 and 0.9892, respectively. The regression model was noted to be very significant (*p* < 0.0001), and no conspicuous Lack of Fit (*p* > 0.05) was presented, indicating that the model fitting degree was good and the test error was small. Hence, the model can be applied to predict the antibacterial effect of CA on the growth of *Y. enterocolitica* in raw pork and *E. sakazakii* in skim milk. The variance test of the regression equation showed that the interaction between temperature and CA concentration had a significant impact on the antibacterial effect of CA on *Y. enterocolitica* and *E. sakazakii* (*X*_2_*X*_3_, *p* < 0.0001). In addition, temperature had no significant effect on the bacterial number of *Y. enterocolitica* within 12–24 h (*X*_2_, *p* > 0.05). Furthermore, the interaction between treatment time and CA concentration (*X*_1_*X*_3_, *p* > 0.05) had no significant influence on *Y. enterocolitica* and *E. sakazakii* growth inhibition.

**Figure 7:**
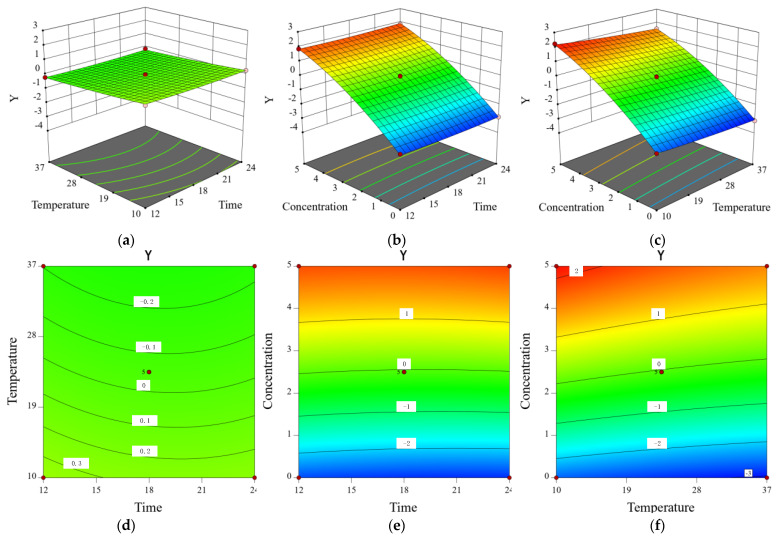
The contour plots showing the effect of interactions between (**a**) storage time and storage temperature, (**b**) storage time and CA concentration, and (**c**) storage temperature and CA concentration on *Y. enterocolitica* growth in raw pork. The response surface plots showing the effect of interactions between (**d**) storage time and storage temperature, (**e**) storage time and CA concentration, and (**f**) storage temperature and CA concentration on *Y. enterocolitica* growth in raw pork.

**Figure 8:**
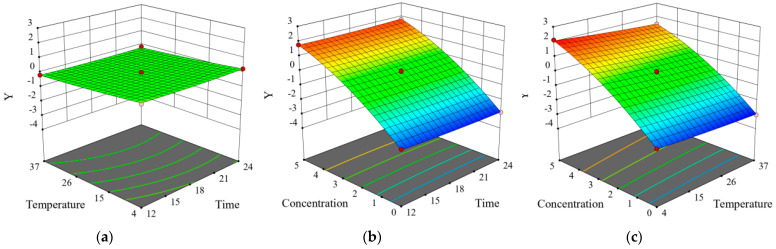

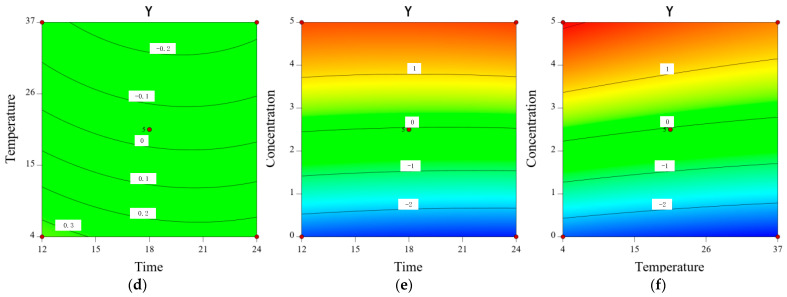
The contour plots showing the effect of interactions between (**a**) storage time and storage temperature, (**b**) storage time and CA concentration, and (**c**) storage temperature and CA concentration on *E. sakazakii* growth in skim milk. The response surface plots showing the effect of interactions between (**d**) storage time and storage temperature, (**e**) storage time and CA concentration, and (**f**) storage temperature and CA concentration on *E. sakazakii* growth in skim milk.

The 3D response surface plot and contour plot (Figure 7 and Figure 8) intuitively displayed the impact of the three factors on the response value and trend of change. The CA of MIC was shown to basically inhibit the increase in *Y. enterocolitica* in raw pork and *E. sakazakii* in skim milk, which further verified the inhibitory effect of CA on *Y. enterocolitica* and *E. sakazakii* in food systems. Similarly, Shan et al. reported that oregano, clove, pomegranate peel, and grape seed extracts can be used as aerobic packaging to inhibit foodborne pathogens (*L. monocytogenes*, *Salmonella enteritidis*, and *S. aureus*) in pork at 20 °C [34].

## 3. Materials and Methods

### 3.1. Reagents

Chlorogenic acid (HPLC ≥ 98%) was purchased from Biotech Bioengineering (Shanghai) Co., Ltd. (Shanghai, China) and dissolved in Luria-Bertani (LB) medium and phosphate-buffered saline (PBS) containing 2% (*v/v*) ethanol in order to prepare different concentrations of CA. All other reagents were of analytical grade.

### 3.2. Bacterial Strains and Culture Conditions

*Yersinia enterocolitica* (BNCC 108930) and *Enterobacter sakazakii* (BNCC 186080) were purchased from BeNa Culture Collection (BNCC, Beijing, China) and cultured in LB medium. *Y. enterocolitica* and *E. sakazaki*i frozen at −80 °C were activated on a LB plate, after which a single colony was inoculated into LB medium and cultured overnight. In order to obtain a fresh bacterial suspension, the overnight cultured bacterial suspension was transferred to a 100 mL LB medium and cultivated to the logarithmic phase (approximately 1 × 10^8^ CFU/mL).

### 3.3. Determination of MIC

The MIC was measured via microdilution in 96-well microtiter plates [35]. The preparation of the bacterial suspension was described in Section 2.2, and the optical density (OD) of the bacterial suspension at 600 nm was adjusted to 0.5. The OD_600_ of 0.5 corresponded to approximately 1 × 10^8^ colony forming units (CFU)/mL. Then, 190 μL of bacterial suspension and 10 μL of CA were added into the 96-well plate (Nunc, Copenhagen, Denmark). Afterward, the mixture was cultured at 37 °C for 8 h. Finally, the OD_600_ was monitored with a multimode reader (Synergy H1, BioTek, Winooski, VT, USA). The final concentrations of CA were 10, 5, 2.5, 1.25, 0.625, 0.3125, and 0.15625 mg/mL. The LB-containing 2% ethanol group and 2.5 mg/mL ampicillin group were labeled as the negative control group and positive control group, respectively. The MIC was the lowest CA concentration without visible bacterial growth.

### 3.4. Bacterial Growth Curve

The antibacterial activity was reflected by the determination of the bacterial growth curve according to Kang et al. with a few modifications [36]. Briefly, 190 μL of bacterial suspension with an OD_600_ of 0.5 and 10 μL of CA were added into the 96-well plate (Nunc, Copenhagen, Denmark). Then, the mixture was cultured at 37 °C for 24 h. Finally, the OD_600_ was monitored with a multimode reader (Synergy H1, BioTek, Winooski, VT, USA) every 1 h. The final concentrations of CA were 10, 5, 2.5, 1.25, 0.625, 0.3125, 0.15625, and 0.0781 mg/mL. The LB-containing 2% ethanol group and 2.5 mg/mL ampicillin group were labeled as the negative control group and positive control group, respectively.

### 3.5. Determination of Membrane Potential

The experiment was carried out according to Wu et al. with some modifications [37]. The preparation of the bacterial suspension was described in Section 2.2. The supernatant of the bacterial suspension was removed by centrifugation (4000× *g*, 10 min, 4 °C), and the bacteria were washed twice with PBS and suspended in PBS. CA was then added to the bacterial suspension with an OD_600_ of 0.5 to achieve final concentrations of 0, MIC, and 2 MIC. Next, the sample was cultured at 37 °C for 2 h, and 200 μL of bacterial suspension was added to the black, opaque 96-well microtiter plates (Nunc, Copenhagen, Denmark), while 1 mM of fluorescent probe bis-(1,3-dibutylbarbituric acid) trimethine oxonol (DiBAC4(3); Molecular Probes, Sigma, Louis, MO, USA) was added to the sample. After incubating at 37 °C for 15 min in the dark, the fluorescence intensity was measured by a multimode reader (Synergy H1, BioTek, Winooski, VT, USA). The excitation wavelength was 492 nm, and the emission wavelength was 515 nm.

### 3.6. Measurement of Intracellular ATP Concentrations

The intracellular ATP concentration was determined through the method put forward by Han et al. [38], and preparation of the bacterial suspension was described in Section 2.2. The supernatant of the bacterial suspension was removed by centrifugation (4000× *g*, 10 min, 4 °C), and the bacteria were washed twice with PBS and suspended in PBS. CA was then added to the bacterial suspension with an OD_600_ of 0.5 to achieve final concentrations of 0, MIC, and 2 MIC. Then, the sample was cultured at 37 °C for 2 h, and the bacteria were washed 3 times with PBS and collected via centrifugation (4000× *g*, 10 min, 4 °C). Afterward, 100 μL of cell lysate buffer was added to the sample in order to lyse the cell, after which the supernatant was collected via centrifugation (4000× *g*, 10 min, 4 °C) and stored on ice to prevent ATP degradation. The content of intracellular ATP was then determined using an ATP assay kit according to the manual’s instructions (Beyotime Bioengineering Institute, Shanghai, China). Specifically, 100 μL of detection working solution was added to the black 96-well microplate (Nunc, Copenhagen, Denmark), after 10 min, and the background ATP in the microplate was eliminated. Then, 20 μL of supernatant was added to the 96-well microplate. Finally, the chemiluminescence intensity of the sample was determined using a multimode reader (Synergy H1, BioTek, Winooski, VT, USA).

### 3.7. Measurement of Intracellular pH Level

pHin was determined according to a modified method put forward by Shi et al. [39], with the preparation of the bacterial suspension described in Section 2.2. The supernatant of the bacterial suspension was removed by centrifugation (4000× *g*, 10 min, 4 °C), and the bacteria were washed twice with HEPES buffer solution. Next, 3 mM of fluorescent probe carboxyfluorescein diacetate succinimidyl ester (cFDA-SE; Molecular Probes, Sigma, Louis, MO, USA) was added to the bacterial suspension. After 20 min, the cells were washed twice with PBS, and 10 mM of glucose solution was added and incubated at 37 °C for 30 min to eliminate non-conjugated cFDA-SE. Finally, the cells were washed twice with PBS and suspended in PBS. CA (0, MIC, and 2 MIC) was added to the cell culture and stained by fluorescence in black, opaque 96-well microtiter plates (Nunc, Copenhagen, Denmark) for 2 h. The fluorescence intensity was measured by a multimode reader (Synergy H1, BioTek, Winooski, VT, USA). The excitation wavelength was 490 nm and 440 nm, and the emission wavelength was 520 nm.

Calibration curves were determined by the cFDA-SE-loaded cells with different pH buffers. The pH buffer was prepared using glycine (50 mM), potassium chloride (50 mM), citric acid (50 mM), and sodium dihydrogen phosphate (50 mM). The pH (3, 4, 5, 6, 7, 8, 9, and 10) was then adjusted with NaOH/HCL. The bacteria were suspended in different pH buffers, and valinomycin was added to equilibrate the pHin and pHout. Finally, the fluorescence intensity was determined, and the pHin of the cells was evaluated according to the ratio of the fluorescence signal of the pH-sensitive wavelength (490 nm) and pH-insensitive wavelength (440 nm).

### 3.8. Confocal Laser Scanning Microscopy Analysis

Membrane permeability and dynamic changes were evaluated using the LIVE/DEAD BacLight™ Bacterial Viability Kit (Molecular Probes, Thermo Fisher, Waltham, MA, USA) according to the procedure put forward by Du et al. with slight modifications [40]. The preparation of the bacterial suspension was described in Section 2.2. The supernatant of the bacterial suspension was removed by centrifugation (4000× *g*, 10 min, 4 °C), and the bacteria were washed twice with normal saline and suspended in normal saline. CA was added to the bacterial suspension with an OD_600_ of 0.5 to achieve final concentrations of 0, MIC, and 2 MIC, respectively. Then, the sample was cultured at 37 °C for 2 h, and the bacterial suspension was washed 3 times with normal saline and suspended in normal saline. Next, 3 μL of the 2× staining solution (SYTO9/PI) was added to 1 mL of the bacterial suspension. After incubating at 37 °C for 15 min in the dark, the bacteria were washed 3 times with normal saline and suspended in 100 μL of normal saline. Finally, 2 μL of bacterial suspension was transferred to a glass slide and observed by CLSM (LSM800, Carl Zeiss, Yarra, Germany).

### 3.9. Field Emission Gun Scanning Electron Microscope Analysis

The effects of CA on the morphology of *Y. enterocolitica* and *E. sakazakii* were determined using FEGSEM [41]. The preparation of the bacterial suspension was carried out according to Section 2.2. The supernatant of the bacterial suspension was removed by centrifugation (4000× *g*, 10 min, 4 °C), and the bacteria were washed twice with PBS and suspended in PBS. CA was added to the bacterial suspension with an OD_600_ of 0.5 to achieve final concentrations of 0, MIC, and 2 MIC. The sample was then cultured at 37 °C for 2 h, and the cells were washed 3 times with PBS and collected by centrifugation (4000× *g*, 10 min, 4 °C). They were then fixed with 2.5% glutaraldehyde solution overnight at 4 °C. Then, the bacteria were collected via centrifugation (4000× *g*, 10 min, 4 °C) and dehydrated with a graded series of ethanol (30%, 50%, 70%, 90%, and 100%) for 10 min each. Finally, the bacteria were incubated with isoamyl acetate for 30 min and collected by centrifugation (4000× *g*, 10 min, 4 °C). The bacteria were then dried through freeze-drying, and the dried sample was sprayed with gold on the FEGSEM support. The morphology of the cells was observed using FEGSEM (MLA 650, FEI, Hillsboro, OR, USA).

### 3.10. Modeling the Inhibitory Effect of Chlorogenic Acid on the Growth of Y. enterocolitica in Raw Pork and E. sakazalii in Skim Milk

Frozen pork was purchased from a local supermarket and transported to the laboratory. The raw pork was aseptically cut into small pieces (approximately 1.5 cm × 1.5 cm × 1.5 cm) and exposed to ultraviolet light for 30 min to reduce the presence of other contaminants. A suspension of *Y. enterocolitica* was added to the 10 g raw pork sample in order to artificially contaminate the raw pork, with the final bacterial concentration being 10^7^ CFU/g [42]. Skim milk was purchased from a local supermarket. A suspension of *E. sakazakii* bacteria was added to the 10 mL skim milk sample to artificially contaminate the skim milk, with the final bacterial concentration being 10^7^ CFU/mL [43]. The samples were then divided into three groups, the control group (without antibacterial drugs), sample group treated with CA at MIC, and sample group treated with CA at 2 MIC.

### 3.11. Statistical Analysis

All experimental results were analyzed using the SPSS software (SPSS 8.0 for Windows). All data were expressed as mean ± standard deviation (*n* = 3). Analysis of variance (ANOVA) was carried out to determine any significant differences (*p* ≤ 0.01).

## 4. Conclusions

Within the food industry, the application of plant-derived preservatives has received intensive attention. This study demonstrated the effective antibacterial activity of CA against *Y. enterocolitica* and *E. sakazakii* by increasing the permeability of the cell membrane, destroying the integrity of the cell membrane, and causing the leakage of cell contents. Response surface methodology analysis illustrated that CA can be used to inhibit the growth of *Y. enterocolitica* in raw pork and *E. sakazakii* in skim milk. Therefore, CA, as a natural antibacterial preservative, possesses broad application prospects within the food industry.

## Figures and Tables

**Table 1:** The design and response of the Box–Behnken experiment of CA against the growth of *Y. enterocolitica*.

Trial	*X*_1_ (h)	*X*_2_ (°C)	*X*_3_ (mg/mL)	*Y* (SD)
1	12	37	2.5	−0.17(0.02)
2	18	20.5	2.5	−0.07(0.06)
3	12	4	2.5	0.29(0.03)
4	18	20.5	2.5	−0.08(0.05)
5	18	20.5	2.5	−0.04(0.02)
6	24	4	2.5	0.26(0.04)
7	12	20.5	5	1.77(0.03)
8	12	20.5	0	−2.6(0.06)
9	18	37	0	−3.01(0.05)
10	24	20.5	0	−2.87(0.02)
11	18	4	0	−2.54(0.04)
12	18	20.5	2.5	0.03(0.03)
13	18	37	5	1.46(0.05)
14	18	4	5	2.13(0.06)
15	24	20.5	5	1.76(0.03)
16	24	37	2.5	−0.16(0.05)
17	18	20.5	2.5	−0.05(0.02)

**Table 2:** The design and response of the Box–Behnken experiment of CA against the growth of *E. Sakazakii*.

Trial	*X*_1_ (h)	*X*_2_ (°C)	*X*_3_ (mg/mL)	*Y* (SD)
1	18	23.5	2.5	−0.09(0.07)
2	24	23.5	0	−2.89(0.02)
3	18	10	5	2.24(0.04)
4	24	23.5	5	1.83(0.05)
5	12	37	2.5	−0.2(0.02)
6	12	10	2.5	0.3(0.03)
7	12	23.5	0	−2.66(0.05)
8	18	23.5	2.5	−0.09(0.02)
9	12	23.5	5	1.85(0.04)
10	18	37	0	−3.12(0.03)
11	18	23.5	2.5	−0.07(0.06)
12	18	23.5	2.5	0.01(0.03)
13	24	10	2.5	0.29(0.07)
14	24	37	2.5	−0.15(0.04)
15	18	10	0	−2.58(0.02)
16	18	37	5	1.53(0.03)
17	18	23.5	2.5	−0.02(0.05)

**Table 3:** Analysis of Variance (ANOVA) of the regression equation of CA against the growth of *Y. enterocolitica*.

Source	Sum of Squares	df	Mean Squares	F-Value	*p*-Value
Model	42.71	9	4.75	1238.22	<0.0001
*X*_1_	0.0113	1	0.0113	2.94	0.1304
*X*_2_	0.5100	1	0.5100	133.07	<0.0001
*X*_3_	41.13	1	41.13	10,731.54	<0.0001
*X*_1_ *X*_2_	0.0004	1	0.0004	0.1044	0.7561
*X*_1_ *X*_3_	0.0169	1	0.0169	4.41	0.0739
*X*_2_ *X*_3_	0.0100	1	0.0100	2.61	0.1503
*X*_1_^2^	0.0110	1	0.0110	2.86	0.1348
*X*_2_^3^	0.0089	1	0.0089	2.32	0.1712
*X*_3_^2^	1.03	1	1.03	268.08	<0.0001
Residual	0.0268	7	0.0038		
Lack of Fit	0.0194	3	0.0065	3.45	0.1314
Pure Error	0.0075	4	0.0019		
Cor Total	42.74	16			

**Table 4:** Analysis of Variance (ANOVA) of the regression equation of CA against the growth of *E. sakazakii*.

Source	Sum of Squares	df	Mean Squares	F-Value	*p*-Value
Model	45.31	9	5.03	932.19	<0.0001
*X*_1_	0.0055	1	0.0055	1.02	0.3460
*X*_2_	0.5995	1	0.5995	111.01	<0.0001
*X*_3_	43.71	1	43.71	8093.61	<0.0001
*X*_1_ *X*_2_	0.0009	1	0.0009	0.1666	0.6953
*X*_1_ *X*_3_	0.0110	1	0.0110	2.04	0.1961
*X*_2_ *X*_3_	0.0072	1	0.0072	1.34	0.2854
*X*_1_^2^	0.0170	1	0.0170	3.14	0.1195
*X*_2_^3^	0.0099	1	0.0099	1.83	0.2178
*X*_3_^2^	0.9661	1	0.9661	178.88	<0.0001
Residual	0.0378	7	0.0054		
Lack of Fit	0.0297	3	0.0099	4.91	0.0793
Pure Error	0.0081	4	0.0020		
Cor Total	45.35	16			

## Data Availability

The data that support the findings of this study are available on request from the corresponding author.

## Sample Availability

Samples of the compounds are not available from the authors.
